# Examination of coal combustion management sites for microbiological and chemical signatures of groundwater impacts

**DOI:** 10.3389/fmicb.2025.1593892

**Published:** 2025-05-23

**Authors:** Christopher E. Bagwell, Josué A. Rodríguez-Ramos, Sabrina Hoyle, Shelby Phillips, Frederick D. Day-Lewis, Bruce Hensel

**Affiliations:** ^1^Environmental and Biological Sciences Directorate, Pacific Northwest National Laboratory, Richland, WA, United States; ^2^Energy and Environment Directorate, Pacific Northwest National Laboratory, Richland, WA, United States; ^3^EPRI, Groundwater and Land Management Program, Palo Alto, CA, United States

**Keywords:** coal combustion products (CCP), microbial community, amplicon sequencing, signatures, groundwater–surface water interactions

## Abstract

Coal combustion accounts for 40% of the world’s electricity and generates more than a billion tons of coal combustion products (CCP) annually, half of which end up in landfills and impoundments. CCP contain mixtures of chemicals that can be mobile in the environment and impact the quality of surface water and potable groundwater. In this investigation, water samples from 14 coal combustion management sites across 4 physiographic regions in the United States, paired with background and down-gradient groundwater samples, were analyzed for water chemistry and microbiology. The objective was to determine if microbiology data alone, or supported by chemistry data, could reliably differentiate source waters and identify sites where CCP is known or expected to be influencing groundwater. Two percent of the total amplicons showed genus level conservation across CCP management sites, regions, and sample types; corresponding to ubiquitous, facultatively aerobic proteobacterial taxa that are generally recognized for the potential to respire using different terminal electron acceptors. Ordination plots did not reveal significant differences (*p* > 0.05) in 16S rRNA gene amplicon diversity by CCP management site, water sample types, or physiographic regions. Contrastingly, chemistry distinguished sample types by standard water quality metrics (total dissolved solids, Ca:SO_4_ ratio), alkali earth metals (K, Na, Li), selenium, boron, and fluoride. A focused evaluation of 16S rRNA gene amplicons for a subset of CCP management sites revealed microbiological features and chemical drivers (F, Ca, temperature) that positively identified the single CCP management site confirmed to have groundwater impacted by CCP leachate. At this site, 9 genera (>0.5% relative abundance) were exclusive to CCP porewater and downgradient groundwater. Inferred metabolisms for these taxa indicates potential for N and S biogeochemical transformations and 1-C metabolism that are consistent with a reducing environment, as evidenced by low ORP and depleted SO_4_^2−^. This research contributes to a growing understanding of conditions where these data types, analyses, and interpretation methods could be applied for distinguishing influence from CCP on the surrounding environment, as well as practical limitations.

## Introduction

Society’s reliance on burning coal as a prime source of power and heat has declined, but coal remains an important fossil fuel for electric power generation. Coal combustion contributes approximately 35% electricity generation worldwide with the largest consumers of coal being the United States (16%), China (53%), and India (72%) (Wang et al., 2023). The inherent properties and ranking of coal types are determined by the geological environment and the extent of diagenesis and metamorphism processes that contributed to the formation of the coal deposit (Hower et al., 2022). High rank coal (e.g., anthracite), for example, has high carbon content with lower percent volatile components and impurities. By contrast, lower rank coals (e.g., sub-bituminous, lignite) are less energy dense and produce ash containing greater proportions of impurities, some of which can be harmful if released to the environment (Bell et al., 2011). Most fossil fuel combustion waste generated in the U.S. originates from coal-fired power plants. Coal combustion products (hereafter referred to as CCP) include boiler slag or bottom ash (melted minerals), fly ash, and flue gas desulfurization (FGD) residuals. The United States (U.S.) generates upwards of 130 million tons of CCP annually, and approximately 60% of the CCP generated is beneficially recycled into construction materials like concrete, wall board, grit, road base and fill (American Coal Ash Association).^1^ Unused CCPs in the U.S. were historically managed in impoundments or landfills, some of which were constructed before modern waste containment design was implemented. Since 2015 there has been a shift away from wet management in impoundments and toward dry management in landfills that have liners and leachate collection systems. Prior to 2015, regulations for managing CCPs varied by State. Then in 2015 the U.S. Environmental Protection Agency established federal regulations on CCP disposal with the 2015 Coal Combustion Residuals (CCR) Rule (40 CFR Parts 257 and 261, 80 FR 21302), which has been amended several times since (Federal Register: Hazardous and Solid Waste Management System; Disposal of Coal Combustion Residuals From Electric Utilities, 2015). These policy actions specify requirements on electric utilities for coal ash disposal, storage, and environmental monitoring to ensure the protection of groundwater and surface water.

One aspect of environmental monitoring specified in the CCR Rule is that groundwater compliance is conducted in phases. As a first step, statistical comparisons are made for measured concentrations of constituents of concern between downgradient groundwater and background groundwater. A statistically significant exceedance in downgradient groundwater is then evaluated against groundwater protection standards. A statistical exceedance in concentration above these standards can indicate that a release has occurred from the CCP unit, which could initiate corrective actions and additional groundwater monitoring. However, factors unrelated to the CCP unit can also contribute to statistical exceedance, such as sampling error, laboratory error, statistical error, natural variation, and sources of contamination other than the monitored CCP unit (EPRI, 2017a). Therefore, the CCR Rule includes provisions to allow an alternative source demonstration (ASD) be performed to determine likely factors or sources independent of the monitored CCP unit. This flexibility built into the regulatory framework allows for site-specific management of CCP and can avoid costly corrective actions through the implementation of risk-based solutions.

ASDs can have multiple lines of evidence, including water quality data analysis and observations on local hydrogeology, the design of a unit, and the absence or presence of another potential source of contamination (EPRI, 2017a). In some challenging cases, additional lines of evidence are needed, one of which is stable isotopic analysis of CCP leachate components (Harkness et al., 2016; Ruhl et al., 2012; Wang et al., 2023; EPRI, 2017a, 2022). Strontium, for example, is present in coal combustion products and mobile in the environment. Published studies have demonstrated that ^87^Sr/^86^Sr ratio from fly ash leachate could be reliably distinguished against low backgrounds of naturally occurring Sr (EPRI, 2012, 2017b; Hurst et al., 1991; Ruhl et al., 2012). Boron (^11^B) is a proven isotopic signature of CCP that is often enriched and easily mobilized and leachable (EPRI, 2012, 2017b; Ruhl et al., 2012; Ruhl et al., 2014). While isotope enrichment signatures and fractionation patterns can serve as powerful tracers of CCP and reactive transport of constituents in the environment, successful deployment of these tools can require extensive baseline studies to delineate site-specific features and variability before source identification can be attributed.

Another potential line of evidence that has been suggested for ASDs is microbial forensics (EPRI, 2022). However, CCP management sites, and their surrounding environments, have not been extensively studied for distinctive microbiological features (taxonomic or functional) or adaptations that could faithfully indicate contaminant leaching and CCP influence on groundwater. Microbial interactions with metals of environmental importance are well-documented (Haferburg and Kothe, 2007; Li et al., 2024; Ramke and Jeyaraman, 2022; Tian et al., 2024); however, the specific microbial responses to CCP exposure remain largely unexplored. Metals contamination is known to exert selective pressure on microbial communities, resulting in an overall decrease in microbial diversity and abundance (Deonarine et al., 2023; Li et al., 2023; Sun et al., 2024) with coincidental enrichment of metal, and antibiotic, resistant taxa (Gillieatt and Coleman, 2024; Hu et al., 2021; Thomas et al., 2020; Vats et al., 2022). Sun et al. (2024) observed a significant direct correlation between specific microbial taxa (e.g., increased abundance of *Proteobacteria* and *Chloroflexi*, with a concomitant decrease in *Acidobacteria*) and coal-related metals such as Cd, Pb, Cu, Zn; although these elements are not common constituents of concern in groundwater at CCP sites (EPRI, 2021). Indirectly, release of CCP leachate into subsurface environments can change the groundwater redox status by stimulating anaerobic respiration of Fe_3_^+^ and SO_4_^2−^ (Deonarine et al., 2023). There is ample evidence to demonstrate that microbial communities are responsive to environmental assaults; however, the sensitivity and stability of microbiological response indicators (e.g., diversity, relative abundance, key taxa) to CCP exposure in site groundwater is uncertain.

The objective of this research was to evaluate the sensitivity of microbial genomics and DNA sequencing to inform the development of new lines of evidence for ASDs. The integrated application of microbial community sequencing and groundwater chemistry data to characterize the extent of CCP environmental impacts is a new application space. This study investigated a collection of CCP management sites to identify unique chemical, microbiological, or coupled signature(s) that could indicate CCP influence of groundwater. The study aimed to (1) determine if conserved microbiological and chemical features were identifiable from different CCP units and (2) assess the utility of these data types for identifying CCP impacted groundwater in a blind study. We hypothesized that these data types collected from diverse CCP management sites could inform on changes in environmental conditions or biogeochemical processes as a response indicator to CCP influence on groundwater quality.

## Materials and methods

### Site selection and sampling

CCP management sites were selected for water sampling to represent a cross-section of CCP management scenarios and background hydrogeologic conditions within the continental United States (U.S.). The sampled sites represented a range of conditions and controlling factors for background groundwater distributed across four physiographic regions of the contiguous U.S., representing a range of hydrogeologic environments and climatic conditions. Water samples were obtained from one CCP management site in the Intermontane Plateaus, five from Appalachian Highlands, eight from the Interior Plains, and one from the Interior Highlands. Most groundwater samples (GW-upgradient and GW-downgradient) were collected within 50 feet below ground surface (bgs) (*n* = 11). Four samples were obtained between 50 and 100 feet bgs, and six samples were collected from depths beyond 100 feet bgs. Additional site selection parameters included factors affecting CCP porewater quality. Most CCP porewater samples originated from coal ash (31 out of 42), though bottom ash (*n* = 3), flue-gas desulfurization (*n* = 6) solids, and mixed residues (*n* = 2) were also represented. Sites include CCPs placed in landfills (*n* = 4) and surface impoundments (*n* = 14) and a distribution of parent coal rankings (bituminous, *n* = 10, to subbituminous, *n* = 3, and blends, *n* = 5) were also captured. A total of 38 CCP porewater, 17 GW-downgradient, and 21 GW-Upgradient were collected, for a total of 76 samples.

Samples were collected by the participating CCP management sites in the fall of 2023 following a sample plan provided by EPRI. The sampling plan detailed sample bottle preparation, sampling order, field filtration, and sample shipment as described below. Other aspects of sample collection followed each site’s standard operating procedure. Prior to sampling, the site’s analytical laboratory provided one to two 1,000-mL pre-labeled acid-cleaned HDPE bottles for each sampling location. One bottle was used for most monitoring wells and two bottles were used for less-turbid sample points such as ponds, leachate collections systems, and certain monitoring wells. The microbiological sample bottles did not receive a preservative. For each groundwater monitoring well and CCP porewater collection point, the samplers first purged a volume of water that was slightly more than the total volume contained in the pump and tubing. Next the sample bottle was rinsed with water from the sample point, and then the bottle was filled without filtering. Water samples for microbiological analysis were shipped to Pacific Northwest National Laboratory (Richland, WA, USA) and held at 4°C for processing. Sampling metadata is available in Supplementary Table 1.

### Water chemistry

CCP management sites also collected samples of the groundwater and CCP porewater for standard water chemistry analysis. These samples were collected during the same event as the microbiological samples, after the microbiological samples had been collected. These samples were distributed to internal or contracted external laboratories for water quality analysis using standard EPA analytical methods.^2^ Inter-laboratory variability was checked by including United States Geological Survey standard water reference samples (SWRS) with shipments to each laboratory. SWRS samples provide for inter-laboratory comparison studies with the use of test samples made with natural-matrix water and spiked with reagent chemicals.^3^ Relative percent difference (RPD) calculations showed that most laboratories returned results within 10% of the most probable value (median) calculated by USGS for the each SWRS sample. Any chemical value that was not present in at least 30 samples was removed (Ag, Br, P, U). Any measurement that was non-detect was replaced with zero, and chemicals that were not collected across all sites were removed given that they would not be comparable for our full site-level analyses (Alkalinity, Oxidation–Reduction Potential, pH, Dissolved Oxygen, Turbidity, Ag, Br, Cl, Cu, Zn, Mn, Ni, Mg, P, U). Water chemistry is available in Supplementary Table 1.

### DNA extraction

The microbiological water samples (1 L) were filtered using Sterivex™ filter units to collect microbial biomass (0.22 μm pore size PES membrane). Cell lysis and DNA extraction utilized the DNeasy PowerWater kit (Qiagen, Düsseldorf, NRW) and DNA quality was quantified using NanoDrop UV–Vis spectrophotometer. Genomic DNA preparations were shipped frozen to by Azenta/GENEWIZ (Azenta US, Inc., South Plainfield, NJ, USA) for 16S rRNA gene sequencing.

### Amplicon sequence generation and processing

16S-EZ rDNA next generation sequencing library preparations and Illumina sequencing were conducted at Azenta Life Sciences (South Plainfield, NJ, USA). Sequencing library was prepared using a 16S rDNA Library Preparation kit (Azenta Life Sciences, South Plainfield, NJ, USA). Briefly, the DNA was used to generate amplicons that cover V3 and V4 hypervariable regions of bacteria and archaeal 16S rDNA. Indexed adapters were added to the ends of the 16S rDNA amplicons by limited cycle PCR. DNA libraries were validated and quantified before loading. The pooled DNA libraries were loaded on an Illumina instrument according to manufacturer’s instructions (Illumina, San Diego, CA, USA). The samples were sequenced using a 2× 250 paired-end (PE) configuration. Image analysis and base calling were conducted by the Illumina Control Software on the Illumina instrument.

16S rRNA amplicon sequencing samples were processed using QIIME2 version 2024.5 (Bolyen et al., 2019). Samples were denoised and quality filtered with DADA2 (Callahan et al., 2016). For specific details regarding quality control commands with QIIME2 please see our data repository.^4^ Sequences that were flagged by GenBank as being potential contaminants and/or having low sequence similarity to known organisms were removed (*n* = 5%) to prevent false positives. All amplicon sequence variants (ASVs) were then taxonomically classified within QIIME2 using the GTDB r220 classifiers downloaded from the qiime2 resources webpage: https://resources.qiime2.org/. Raw counts and taxonomic assignments were then exported and processed in R. All commands for QIIME2 sequence processing can be found on GitHub: https://github.com/jrr-microbio/coal_combustion_products. QIIME2 sample processing metadata is available in Supplementary Table 2. ASV and genera level abundances are available in Supplementary Table 1. Raw sequencing data is available on Zenodo: https://zenodo.org/records/15122667.

### Statistical analyses

To account for uneven sequencing depths, overall counts per each sample were normalized into relative abundance values and used as input for comparisons. To determine the overall composition of ASVs across treatments, we calculated Bray-Curtis dissimilarities using vegan in R^5^ and visualized them using Nonmetric multidimensional scaling (NMDS) with *k* = 2. Statistical differences between the Bray-Curtis dissimilarities of ASV communities and overall chemistry per samples were determined using PERMANOVA with the adonis2 package (Anderson, 2017). Further, a Kruskal-Wallis test using the base R stats package was performed for comparisons of individual chemical values across groups. Statistical comparisons between ASVs and the axes of NMDS were performed with a Spearman Rank correlation as well as an env.fit function from the vegan package. Heatmap of different genera per sample were generated with the pheatmap package.^6^ Note, samples that were labeled as “Ash Pond” and “CCP Porewater” were all treated as if they were the same treatment. Only four of the sites had all 3 sample types (i.e., porewater, upgradient groundwater and downgradient groundwater) and at least 6 replicate samples for statistical assessment of site-level variability. All commands used in R can be found via the R markdown on GitHub: https://github.com/jrr-microbio/coal_combustion_products.

## Results and discussion

### Summary of data collected from CCP management sites

This investigation provides analysis and comparison of microbiological and chemical features of water samples for CCP management sites at a single time point as part of a blind study to identify potential signatures that could indicate CCP influenced groundwater. From the 14 participating CCP management sites, 76 water samples (i.e., Ash Pond Water, CCP Porewater, GW-Downgradient, and GW-Upgradient) (i.e., background GW) were extracted for microbial genomic DNA. Corresponding water chemistry data was curated by removing variables having incomplete measurements across multiple samples, yielding a final table that included 25 different features for all 76 samples. For the CCP management site 16S rRNA gene amplicon dataset, we recovered 25,579,517 reads which were denoised and quality controlled with DADA2 to yield 23,888,770 quality reads. Sample sizes were not uniform across CCP management sites, resulting in an uneven distribution of data (minimum = 10,172 reads, maximum = 712,433 reads, average = 314,325.92 reads).

We note that water samples were received from CCP management sites at different times, resulting in uneven storage durations (30–60 days at 4°C) prior to processing. Variable sample hold time could have differentially influenced microbial community diversity between CCP sites and samples. Many CCP groundwater samples were chemically reduced at the time of collection as indicated by ORP measurements, which could have become more oxidizing during storage (Supplementary Table 1). The impact of sample storage time and conditions on microbial community composition is commonly discussed in the literature. There are numerous examples that demonstrate clear alterations in the microbial community during storage which could obscure characterization measurements or inferred comparisons between samples (Lipus et al., 2019; Rachel and Gieg, 2020). Other examples, though, do show that the impacts of sample storage are not significant (Blekhman et al., 2016; Edwards et al., 2024; Holzhausen et al., 2021). It is challenging to establish a consensus on sample storage that applies equally across different environments, though we might expect the relative influence to be proportional to the magnitude of the disparity in environmental conditions the sample experiences during storage. Consistency in preservation treatments across samples is the recommended practice to ensure the accuracy and intercomparability of data being generated (Rubin et al., 2013). Taking this into consideration, along with the limited temporal and spatial sampling performed, the comparisons conducted and interpretations drawn are limited in scope.

### Microbial ASVs were not significantly different between sample types, region, or across sites, while genera exhibited site-level patterns

**Figure 1:**
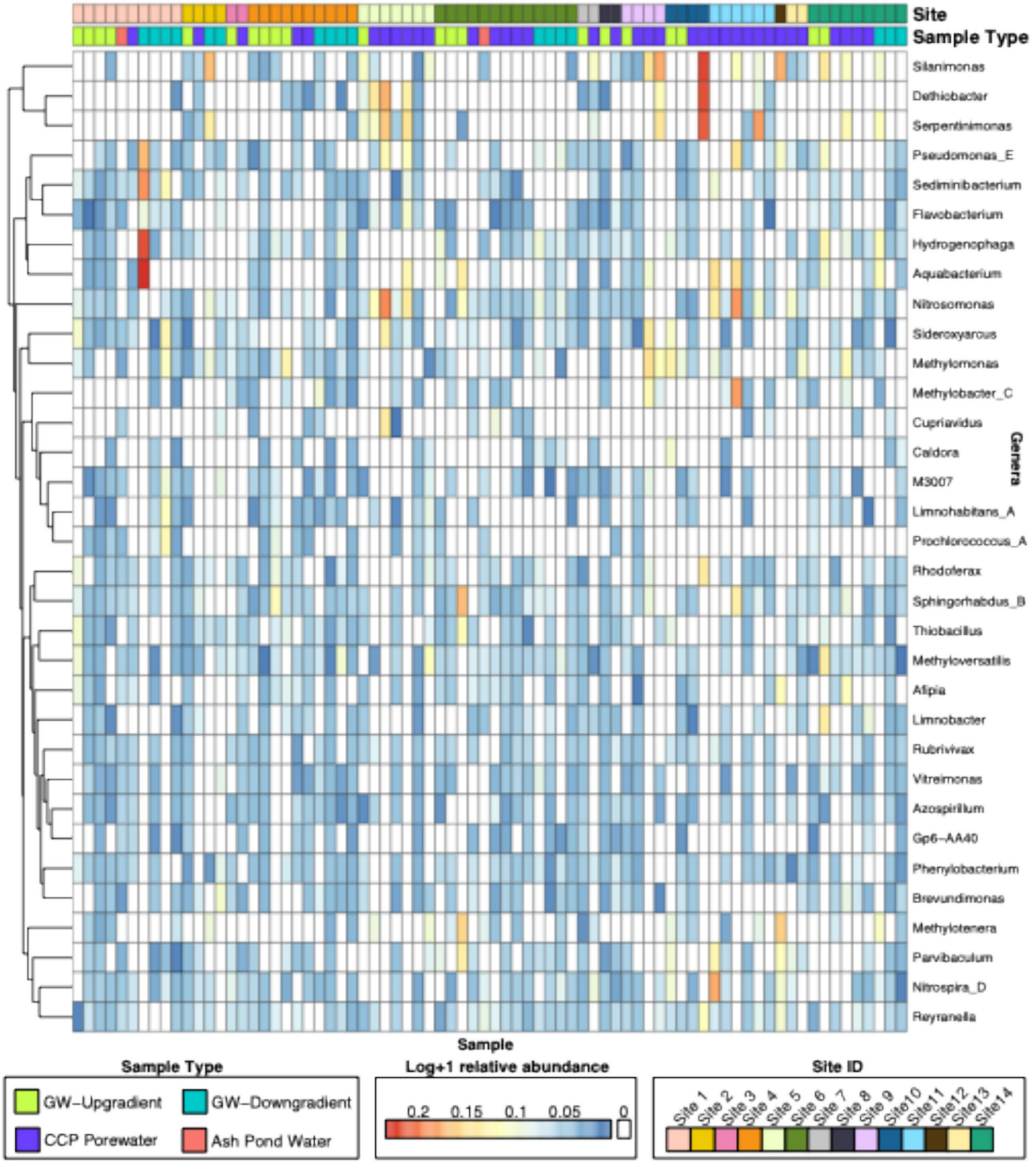
Heatmap of genera-level abundances across all samples. Heatmap denotes log transformed relative abundances of ASVs aggregated at the genera level by GTDB taxonomy. Samples were allowed to cluster by rows, and boxes on the top represent either Site IDs or Sample Types for each sample. Only genera that had greater than or equal to 0.5% of relative abundance are shown.

In total, 48,164 amplicon sequence variants (ASVs) were phylogenetically classified using a QIIME2 pre-trained GTDB classifier (Parks et al., 2022) and aggregated into 3,389 genera, of which only 33 had greater than 0.5% relative abundance across all samples, accounting for 933 ASVs (Figure 1). Overall, neither ASVs nor genera were significantly different between sample types or region (*p* > 0.05) (Supplementary Figures 1, 2). Contrastingly, genera were significantly different by site (Genera: *R*^2^ = 0.19, *p* ≤ 0.05) (Supplementary Figures 1, 2). No overall pattern in amplicon profiles across CCP sample types is not an unexpected finding, as environmental drivers, as well as operational factors (coal type, combustion products, storage unit type and age) of each CCP management site collectively contribute to high variability in the data set. These drivers will have a direct influence on microbial community diversity and abundance in these samples, though the observation of site-level distinctions at the genera level suggests that a more focused investigation of CCP management sites for baseline and anomalous (emergent features) would constrain noise in the data, as well as help with data sparsity which was likely preventing the detection of significant ASV-level community patterns by site (*R*^2^ = 0.18, *p* = 0.08).

### Chemistry significantly varies between sample type, site, and region

**Figure 2:**
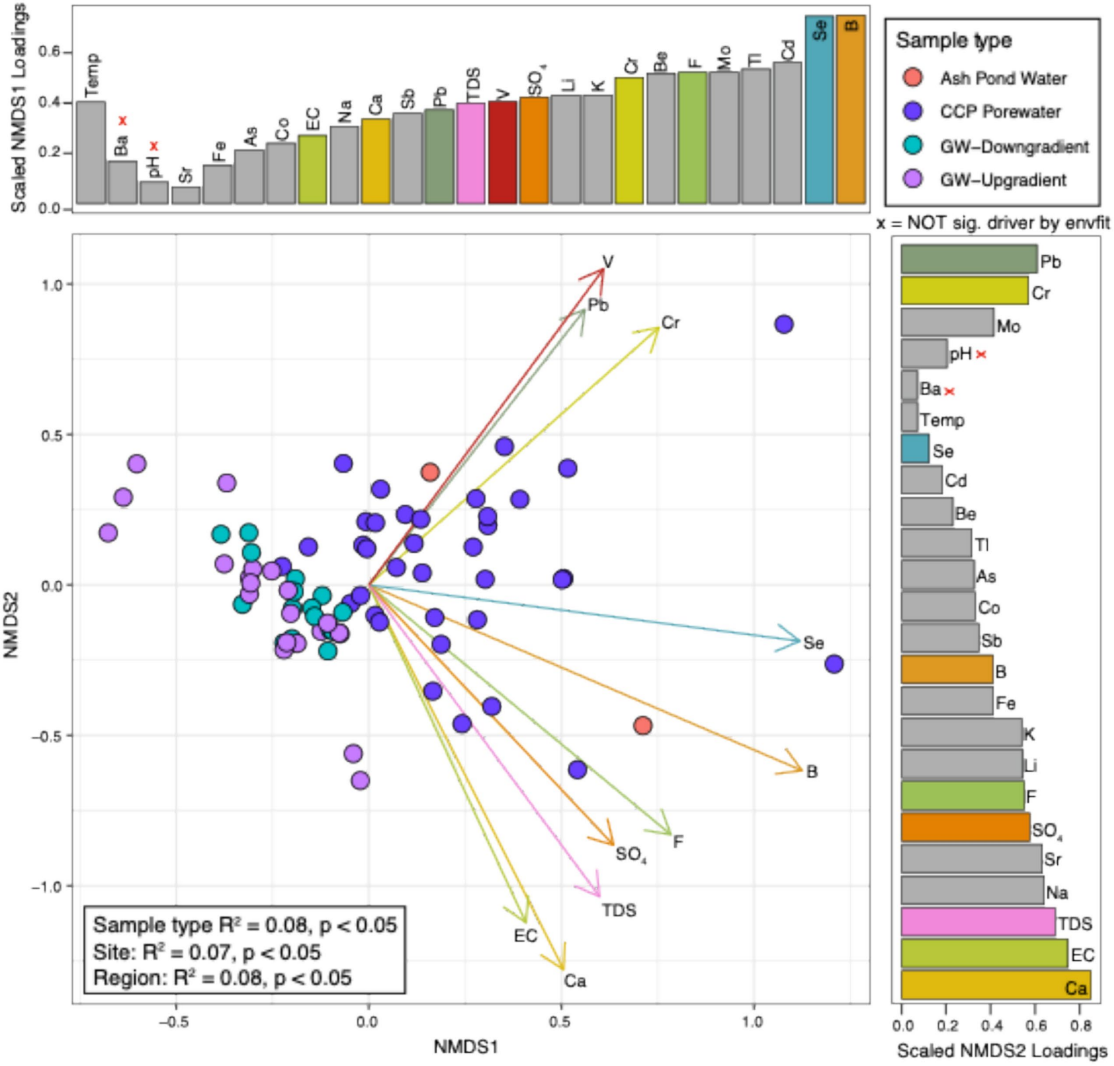
NMDS of water chemistry at CCP management sites highlights significant drivers that distinguish sample types. Non-metric multidimensional scaling (NMDS) figure shows the overall ASVs per each sample (as denoted by dots). Dot colors represent the sample type that each sample belongs to. Loadings (arrows) and bars show an overlay of the chemical drivers of the ordination. The size of bars represents the distance between the end of a loading arrow and the center of the plot. Within each bar plot, the drivers are labeled. The top 10 most significant drivers are shown with solid, numbered arrows within the ordination below. “X” beside chemicals denotes variables that were not significantly different by an env.fit function in R.

Contrary to the microbial community data, water chemistry was significantly different between all variables (e.g., sample type, site, and region) (Figure 2). Overall, the separation of sample type was driven by differences between the CCP porewater and ash pond water relative to the background and down-gradient groundwater chemistry. Specifically, the ordination of CCP porewaters was significantly driven by the following features: calcium (Ca), boron (B), vanadium (V), total dissolved solids (TDS), electrical conductivity (EC), fluoride (F), chromium (Cr), selenium (Se), lead (Pb), and sulfate (SO_4_). As a holistic examination, CCP porewater and ash pond water chemistries were distinct from groundwater (background and down-gradient), as expected, but the combined analysis of microbiology and water chemistry from these site samples failed to yield convincing patterns that could be interpreted as a clear signature of CCP impact across all management sites. As such, these results highlight how multiple lines of evidence provide the clearest picture of the potential impacts of CCPs.

### Microbial communities reveal a high degree of site-endemicity, with a small subset of genera being widespread across sites

Next, we sought to address the overall occupancy of ASVs across our samples. Of the ASVs that were detected in at least one sample (*n* = 38,461), 96% of them (*n* = 36,995) were low occupancy and only present once across all samples. Highlighting site-level heterogeneity, 96% of ASVs (*n* = 37,072) were only identified at individual sites (Supplementary Figure 3A). In contrast, at the genera level only 32% (*n* = 1,071) and 33% (*n* = 1,128) of detectable genera (*n* = 3,389) were present in one sample and a one site, respectively (Supplementary Figure 3B). Nine genera were found to be present across every site: *Reyranella, Phenylobacterium, Nitrospira D, Shewanella, Pseudomonas, UBA9655, Parvibaculum, Afipia, and SYFT01,* and these genera accounted for 936 ASVs (2.4% of total non-zero abundance ASVs). These conserved taxa were not significantly different in abundance between sample types (*p* > 0.05).

Together, these results further suggest that there is significant site-level variability, with a small proportion of ubiquitous microbial genera shared across sites. This outcome is not surprising given all the variability encompassed across the 14 CCP management sites, however, similar trends at genus-level resolution have been documented for soil samples influenced of coal burning power plants in China. Sun et al. (2024) showed that the relative abundance of cosmopolitan fungal genera proved to be more responsive to coal-related environmental factors than 16S rRNA gene amplicons for either bacteria or archaea. Amplicon sequencing is amendable to low sample volumes and biomass yields, as was the case in this study, but the approach provides limited insights into CCP microbial community diversity and functionality (Alteio et al., 2021). Partial gene sequence can identify taxa down to the genus level, possibly the species level, but amplicon data from complex environmental samples invariably contain a moderate percentage of sequence variants that cannot be confidently assigned to known taxa. Further, inference based functional assignments of 16S rRNA gene amplicons (e.g., Langille et al., 2013) are not definitive or reliable as numerous metabolic functions may not be faithfully conserved across taxonomic boundaries (Djemiel et al., 2022; Ortiz-Estrada et al., 2018; Sun et al., 2020).

### Focused evaluation of selected CCP management sites

**Figure 3:**
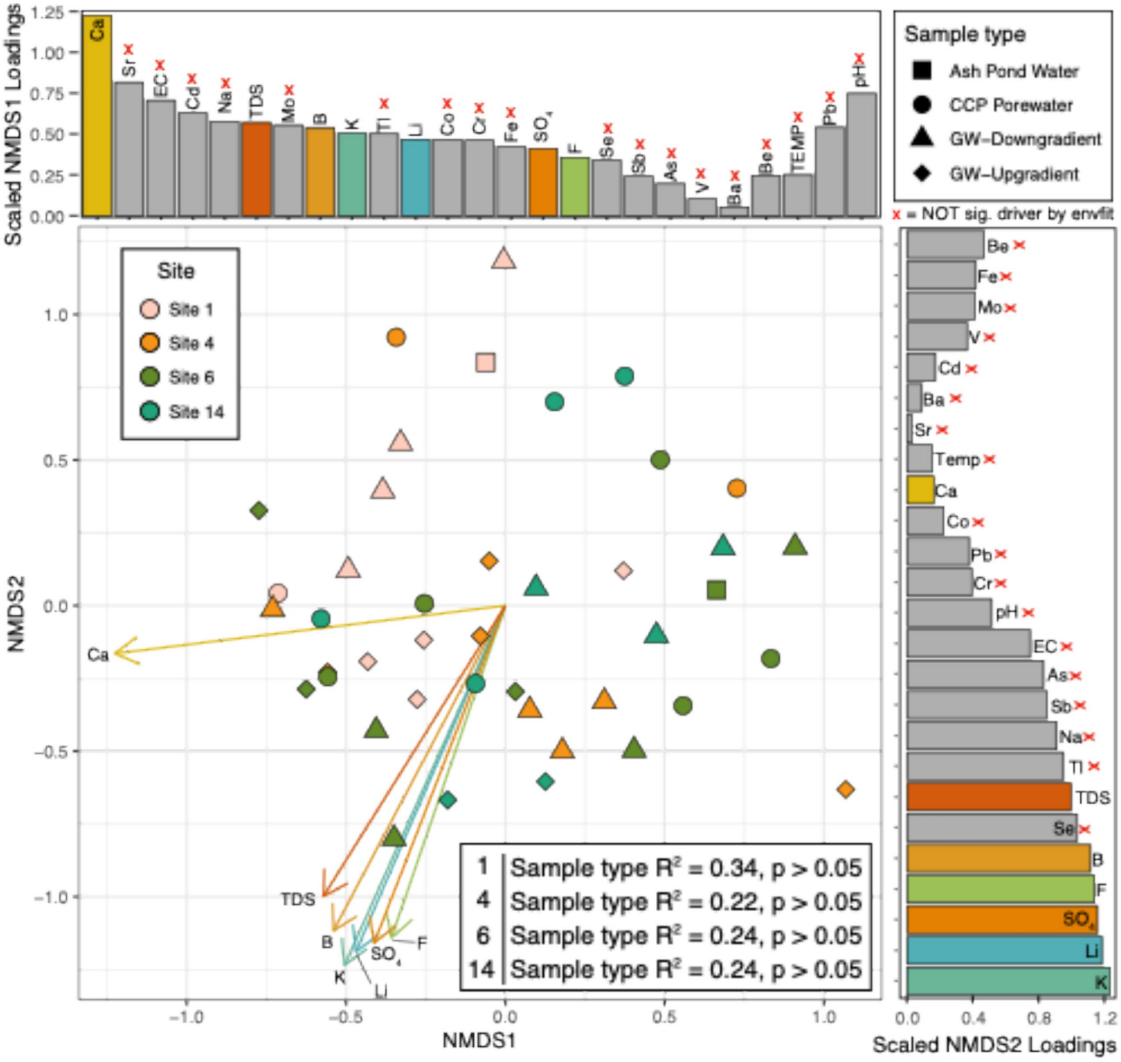
NMDS of ASVs collected from treatment plants highlights chemical drivers of significant differences between sites that had >6 samples. Non-metric multidimensional scaling (NMDS) figure shows the overall abundance of ASVs per each sample (as denoted by dots). Dot colors represent the site samples were collected from. Shapes denote the sample type that each sample belongs to. Loadings (arrows) and bars show an overlay of the chemical drivers of the ordination. The size of bars represents the distance between the end of a loading arrow and the center of the plot. Within each bar plot, the drivers are labeled. The 7 significant drivers are shown with solid, numbered arrows within the ordination below. “X” beside chemicals and gray bars denotes variables that were not significantly different by envfit function in R.

After analysis of ASV communities and chemistry across all sites and samples, further analysis was constrained to four CCP management sites having the most data available to statistically assess the strength of the relationship between sample types, microbiology and water chemistry: i.e., Site 1, Site 4, Site 6, and Site 14 (Figure 3, Supplementary Figure 4, see Methods). Both ASVs and genera in the subset samples were not significantly different between sites, region or sample types (*p* > 0.05). Reflecting these patterns, microbial community richness by either (1) Total number of ASVs (2) Chao index (3) Simpson’s index or (4) Shannon’s index were not statistically significant between sample types (Supplementary Figure 5). Although we did not detect statistical significance between sample types across these subset sites (likely due to low sampling size across all sample variables) we note patterns became apparent within Site 14 between the down-gradient GW and background GW (Figure 3 and Supplementary Figure 4).

Water chemistry was overlayed on the ASV ordinations to identify the most influential chemical drivers at these 4 sites (Figure 3). Across the ordination, the top significant correlations to microbial 16S rRNA amplicon abundance were (in order): potassium (K), lithium (Li), boron (B), calcium (Ca), sulfate (SO_4_), fluoride (F), and total dissolved solids (TDS). These chemical constituents are common in CCP porewaters and, except for potassium, considered as part of an assessment of influence of CCPs on groundwater. All of these chemical parameters also correlated to the separation between CCP porewater or ash pond water samples relative to groundwater samples (Figure 2). Based on these results, we uncovered no evidence of CCP influence on groundwater at sites Site 1, Site 4, and Site 6. This conclusion was consistent with ASDs by the site owners that these three sites were not affecting groundwater quality.

### Microbiological signatures of CCP groundwater influence

**Figure 4:**
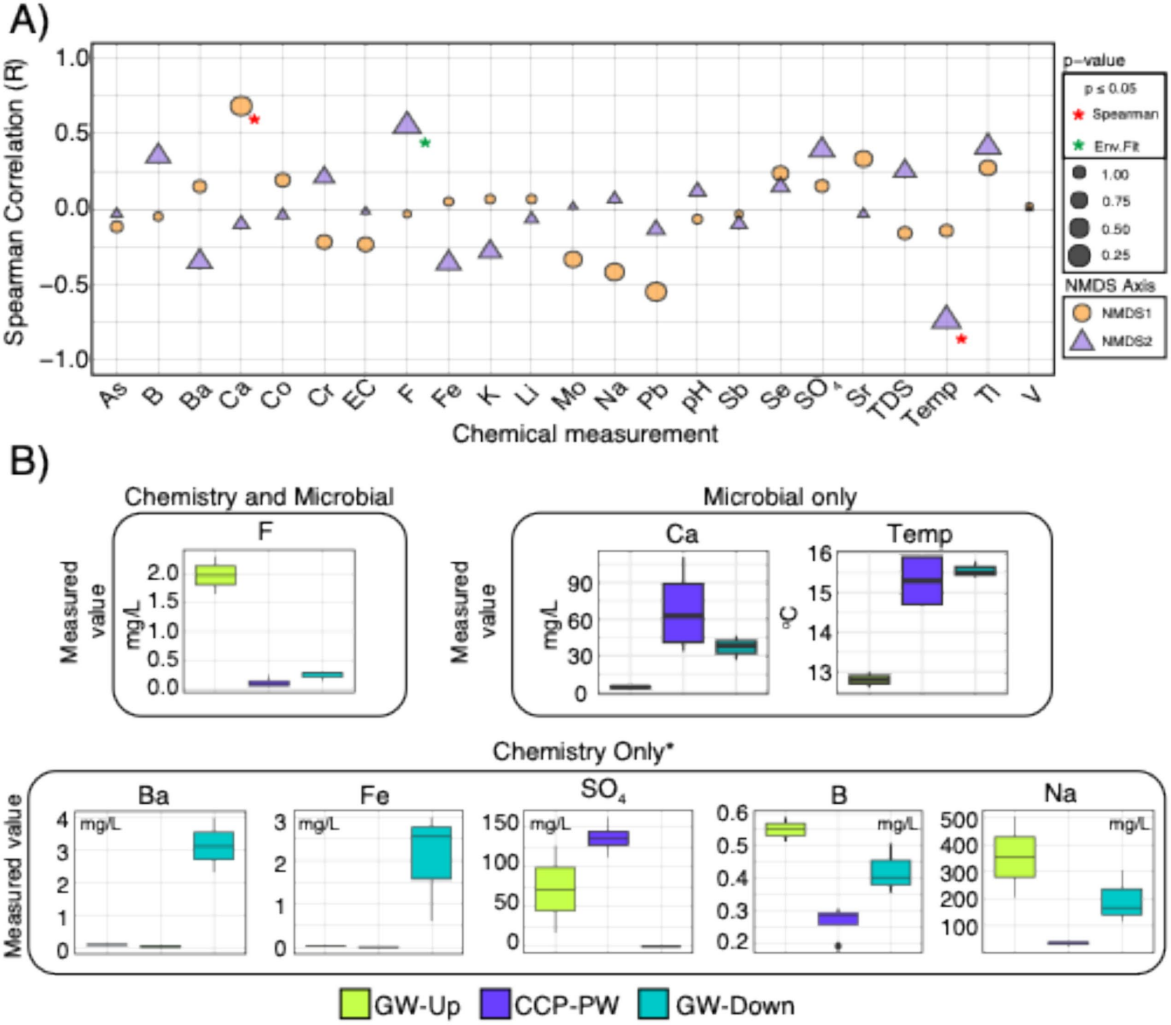
Microbial data indicates Site 14 is potentially being influenced by CCP (Figure 3), and chemical measurements single out calcium, temperature, and fluoride as the driving factors that are significantly correlated with these distinct microbial community patterns. **(A)** Spearman rank correlation results for axes of the NMDS made with subset of ASV abundances for Site 14 (*n* = 9). Colors denote axis (NMDS1 or NMDS2), and size of shapes denote *p*-values. *Y*-axis shows the overall Spearman correlation (R). Significantly correlated chemistry by either (1) Spearman or (2) env.fit are denoted by red or green asterisks, respectively. **(B)** Boxplots denoting chemistry that was flagged as significantly different across our study by Kruskal Wallis test across sample types. Boxes around measurements denote whether it was flagged during analyses of chemical data only, microbial data only, or both. Asterisk (*) on “chemistry only” denotes that there were other chemical values that were significantly different, however, they were excluded because they are only elevated in the CCP, not groundwater. All significant values for chemistry are shown in Supplementary Figure 6.

CCP management Site 14 revealed observable patterns in microbial communities and water chemistry that differentiated background groundwater and down-gradient groundwater (Figure 4). While we advise caution given the limited sample size (*n* = 2 background GW samples), and the lack of *p*-value adjustments of Spearman rank correlations due to these limitations, the overall trends of the NMDS in Supplementary Figure 4 suggest that microbial community amplicon analysis at this site reveals evidence of CCP leachate influenced groundwater.

The water quality parameters that correlated to this ordination pattern in the NMDS2 axis (i.e., the major axis of separation between CCP porewater and background groundwater) was fluoride and temperature, with calcium correlating to the NMDS1 axis (Figure 4). Interestingly, ASVs assigned to 9 genera were absent in background groundwater but were present (>0.5% relative abundance) in CCP porewater and the downgradient groundwater. This result provides reinforcing evidence that CCP leachate has not only influenced groundwater chemistry, but also the microbiology and potentially biogeochemistry at this site. Genera that were exclusive to CCP impacted porewater and downgradient groundwater include *Afipia, Cupriavidus, Methylomonas, Methylotenera, Nitrosomonas, Nitrospira-*D*, Parvibaculum, Serpentinimonas,* and *Sideroxyarcus.* Conserved traits among representative taxa belonging to these genera imply a reducing environment (supported by low ORP and depleted sulfate in downgradient groundwater) conductive to N and S biogeochemical transformations and 1-C metabolism. Nitrogen species are not recognized as constituents of concern at CCP management sites and thus were not measured in this study. Nonetheless, these microbiology data indicate the capacity for complete nitrification metabolism by *Nitrosomonas* (NH_4_^+^ → NO_2_^−^) and *Nitrospira-*D (NO_2_^−^ → NO_3_^−^) (Krüger et al., 2023), and denitrification by *Afipia* (Green et al., 2010). *Parvibaculum* is recognized for metal metabolism and resistance, along with *Cupriavidus* (e.g., von Rozycki and Nies, 2009), as well as the ability to degrade hydrocarbons (Li C. et al., 2023). Potential 1-C substrate sources supporting *Methylomonas* and *Methylotenera* methylotrophy could include methyl sulfates (Richter et al., 2025), generated and concentrated during coal combustion (Lee et al., 1980), and methane, produced within the disposal impoundment or subsurface (Wang et al., 2022). No measurements of soil gases or organic content was measured as part of this study. We caution against overinterpreting these inference-based conclusions. Additional measurements are needed to support a robust microbiological and biogeochemical evaluation of groundwater under the influence of CCP leachate.

Not all the chemistry parameters that correlated to the microbial ASV ordinations (fluoride, calcium, and temperature) of Site 14 were also significantly different by chemical measurements alone (e.g., calcium and temperature). While multiple additional chemistry data within Site 14 were also significantly different across sample types (Supplementary Figure 6), we focused on barium, iron, sulfate, sodium, fluoride, and boron because they were notably different between background and downgradient groundwater, suggesting potential for CCP impacts (Bell et al., 2011; Senior et al., 2020). Barium and fluoride are ubiquitous trace elements in coal and combustion products, and F is typically found to be more abundant than Ba (Vejahati et al., 2010). At this site, fluoride concentration in background groundwater (GW-Upgradient) was significantly elevated relative to Ba (*F* = 1.98 mg/L, Ba = 0.11 mg/L), and Ba > F in downgradient groundwater (*F* = 0.23 mg/L, Ba = 3.15 mg/L). Barium and fluoride concentrations in CCP porewater were lower than background and downgradient groundwater (Figure 4). This concentration profile measured for fluoride was consistent with that of sodium and boron. We do not have a full understanding of this site and its hydrogeology, though it is plausible that the groundwater wells at this site are hydrologically disconnected, or this discrepancy could be a phenomenon of groundwater mixing. Iron levels were elevated in downgradient groundwater (average = 2.0 mg/L), and sulfate concentrations were depleted (average = 0.0 mg/L) in downgradient groundwater despite relatively high concentrations in background groundwater (average 72.05 mg/L) and CCP porewater (136.75 mg/L). A few of these constituents of concern do exceed groundwater standards (e.g., iron, barium) but the inclusion of microbiology in the context of site chemistry provides more compelling evidence for CCP influenced groundwater and insights into the biogeochemistry of the site. Even with limited sampling conducted, CCP influence of groundwater was indicated in the NMDS of ASV communities by the clustering of CCP porewater and downgradient groundwater, and separation from background groundwater. This conclusion was consistent with the site owner’s interpretation that CCP leachate was having an influence on groundwater quality at this site.

### Summary

This study aimed to investigate the use of microbial amplicon data as a supporting line of evidence in ASDs to determine whether groundwater chemistry is affected by a release of porewater from a CCP unit. Distinct differences in water chemistry were observed between water samples within CCP disposal units and surrounding background water, however other comparisons performed of microbiology and chemistry across all CCP management sites were highly variable, revealing no clear trends. Patterns among sites and samples did begin to emerge, however, when the analysis was constrained to sites having enough data for robust statistical analyses. For a subset of 4 CCP management sites, 16S rRNA gene amplicon sequence analysis, reinforced by site water chemistry data, revealed patterns of CCP influence at a single site confirmed to have impacted groundwater. Key chemical indicators, combined with taxonomy-informed metabolic inferences from 16S rRNA gene amplicon sequencing, provide a reasonable reconstruction of biogeochemical conditions in the downgradient groundwater influenced by CCP. However, confirming this interpretation requires additional data, specifically measurements beyond those routinely collected as part of a groundwater monitoring program at CCP management sites. While these results are promising, amplicon sequencing does have limited utility for detecting subtle microbial community responses likely to accumulate during long-term exposure. Community-level whole genome sequencing and assembly (metagenomics) would enable the resolution of microbial community complexity and dynamics, revealing insights into functional potential and identifying positive selection for adaptations such as metal efflux proteins, cellular defense mechanisms, and lipid transport and metabolism that have been shown crucial to survival in metals impacted environments (Das et al., 2017; Hemme et al., 2010, 2016). The recommendation for future investigations would be to prioritize a more constrained investigation of sites within a physiographic region to minimize variability and increase the likelihood of detecting compositional and functional adaptations of microbial communities to CCP influence. Ultimately, incorporating multiple lines of evidence for assessing contaminant influence on groundwater as a standard practice builds confidence in the effectiveness of current operational and waste disposal practices at CCP management sites. Despite its limited scope, this study demonstrates the potential of an integrated framework to better understand the environmental impacts of CCP leachate on groundwater supplies.

## Data Availability

This Targeted Locus Study project has been deposited at DDBJ/EMBL/GenBank under the BioProject PRJNA1251570/BioSample SAMN48010223/Accession KIXO00000000. The version described in this paper is the first version, KIXO01000000. Supplemental code and data required to run code can be found on GitHub: https://github.com/jrr-microbio/coal_combustion_products. All raw fastq data is publicly available on Zenodo: https://zenodo.org/records/15122667. Quality-controlled fasta sequences for each ASV are available on the GitHub repository: https://github.com/jrr-microbio/coal_combustion_products/blob/main/qiime2/filtered_dna-sequences.fasta.

## Footnotes

^1^ https://acaa-usa.org/

^2^ https://www.epa.gov/cwa-methods

^3^ https://qsb.usgs.gov/srs/srs

^4^ https://github.com/jrr-microbio/coal_combustion_products/tree/main

^5^ https://github.com/vegandevs/vegan

^6^ https://cran.r-project.org/web/packages/pheatmap/index.html

## Supplementary material

SUPPLEMENTARY FIGURE 1 ASV level-NMDS highlight that ASVs are significantly different by site, but not by sample type or region. Multiple NMDS where overall ASV abundance per sample are denoted by circles and are colored by either **(A)** site, **(B)** Region, or **(C)** Sample type. Text box denotes the overall significance by PERMANOVA of each ordination.

SUPPLEMENTARY FIGURE 2 Genera level-NMDS highlight that genera are significantly different by site but not by region nor sample type. Multiple NMDS where overall genera abundance per sample are denoted by circles and are colored by either **(A)** site, **(B)** Region, or **(C)** Sample type. Text box denotes the overall significance by PERMANOVA of each ordination.

SUPPLEMENTARY FIGURE 3 Occupancy of ASVs and genera are mostly found in one site (not necessarily the same site). Bar plots denote occupancy of either **(A)** ASV or **(B)** genera (*y*-axis) and show how many units are present per number of sites (*x*-axis).

SUPPLEMENTARY FIGURE 4 Individual NMDS plots of ASV relative abundance for subset of sites that have enough samples for statistical analyses. Each NMDS represents a single site, and within the site, shapes and colors denote the sample types. Note that upgradient groundwater plots within downgradient groundwater and CCP porewater samples for sites 3586, 17481, and 22354; while it plots separately (to the left side) for site 46001.

SUPPLEMENTARY FIGURE 5 ASV richness metrics across subset of sites that had >6 samples per sample type. Boxes denote the overall richness by either: **(A)** total unique counts of ASVs, **(B)** Shannon’s diversity, **(C)** Simpson’s diversity, **(D)** Chao diversity.

SUPPLEMENTARY FIGURE 6 Chemistry that was significantly different across sample types for Site 14. Box plots denote each significantly different chemical identified by a Kruskal-Wallis test at Site 14. Green box denotes chemistry that was significantly different by both microbial and chemical data. The blue box denotes chemistry that was identified as significant by only chemical data.
