# Supplementary figures and images for: Examination of coal combustion management sites for microbiological and chemical signatures of groundwater impacts
Source: Front Microbiol. 2025 May 23;16:1593892. doi: 10.3389/fmicb.2025.1593892 (PMC12141297; doi:10.3389/fmicb.2025.1593892)

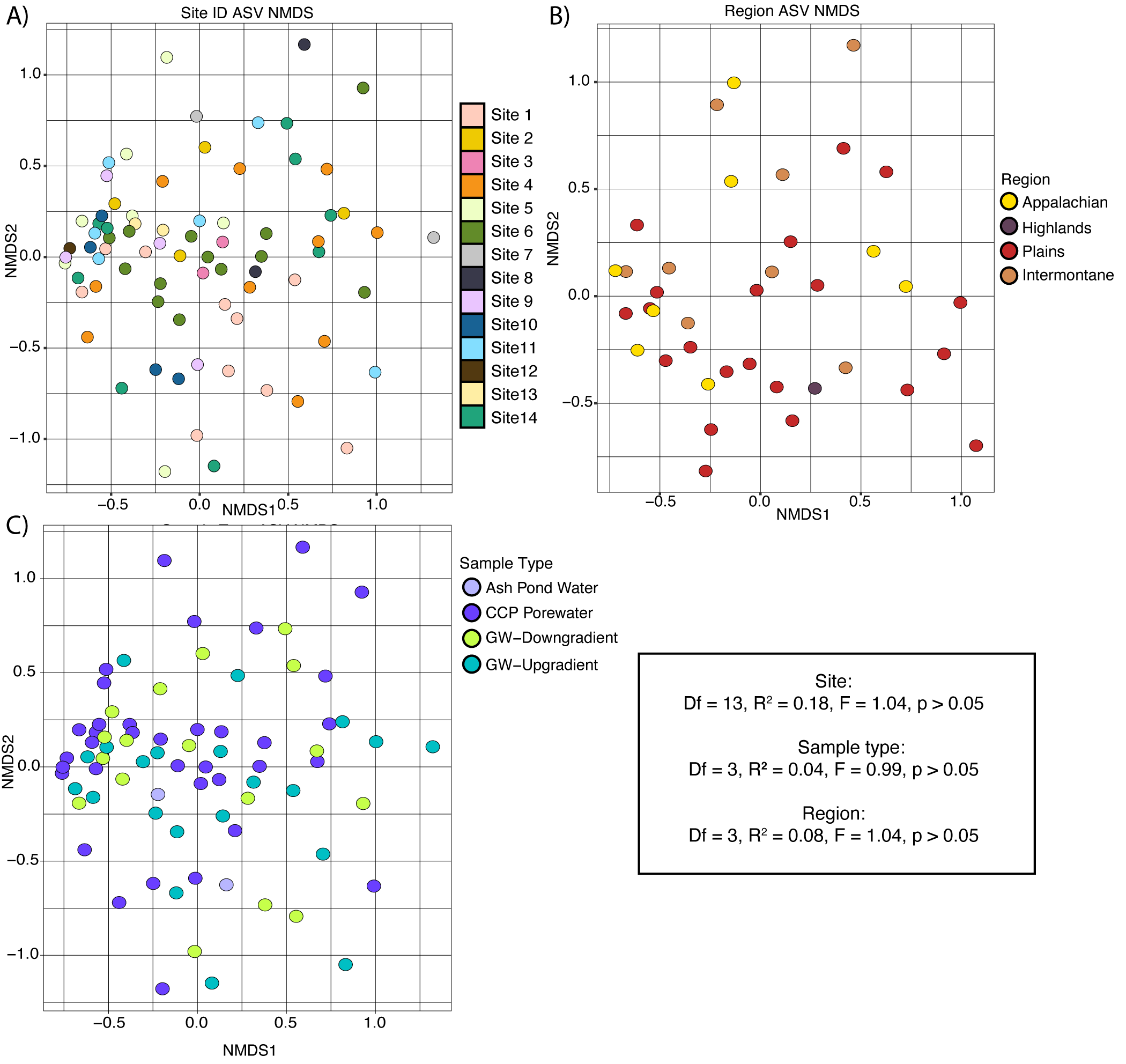

Supplement: SUPPLEMENTARY FIGURE 1 — ASV level-NMDS highlight that ASVs are significantly different by site, but not by sample type or region. Multiple NMDS where overall ASV abundance per sample are denoted by circles and are colored by either (A) site, (B) Region, or (C) Sample type. Text box denotes the overall significance by PERMANOVA of each ordination. [file Image_1.TIF]

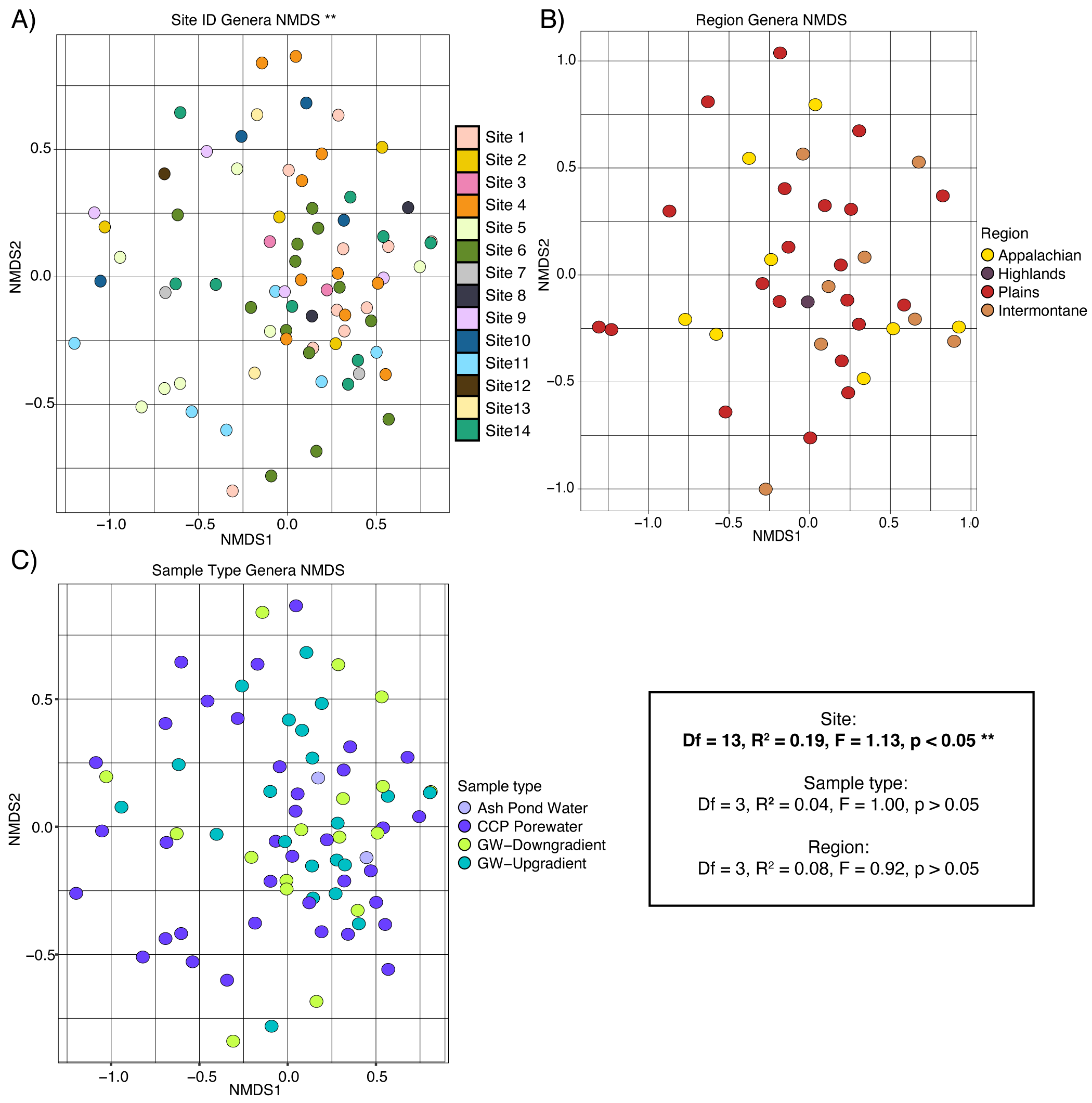

Supplement: SUPPLEMENTARY FIGURE 2 — Genera level-NMDS highlight that genera are significantly different by site but not by region nor sample type. Multiple NMDS where overall genera abundance per sample are denoted by circles and are colored by either (A) site, (B) Region, or (C) Sample type. Text box denotes the overall significance by PERMANOVA of each ordination. [file Image_2.TIF]

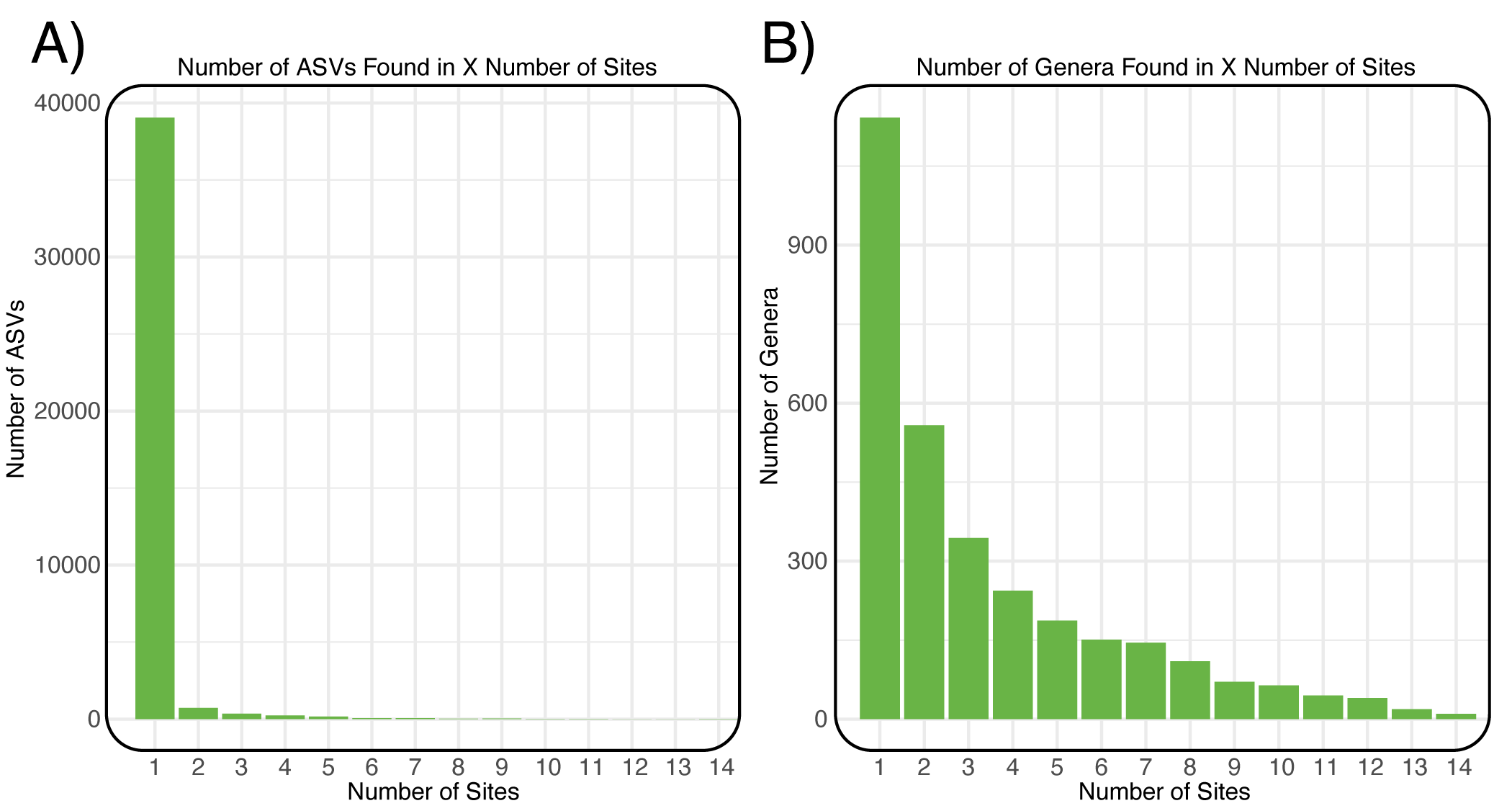

Supplement: SUPPLEMENTARY FIGURE 3 — Occupancy of ASVs and genera are mostly found in one site (not necessarily the same site). Bar plots denote occupancy of either (A) ASV or (B) genera (y-axis) and show how many units are present per number of sites (x-axis). [file Image_3.TIF]

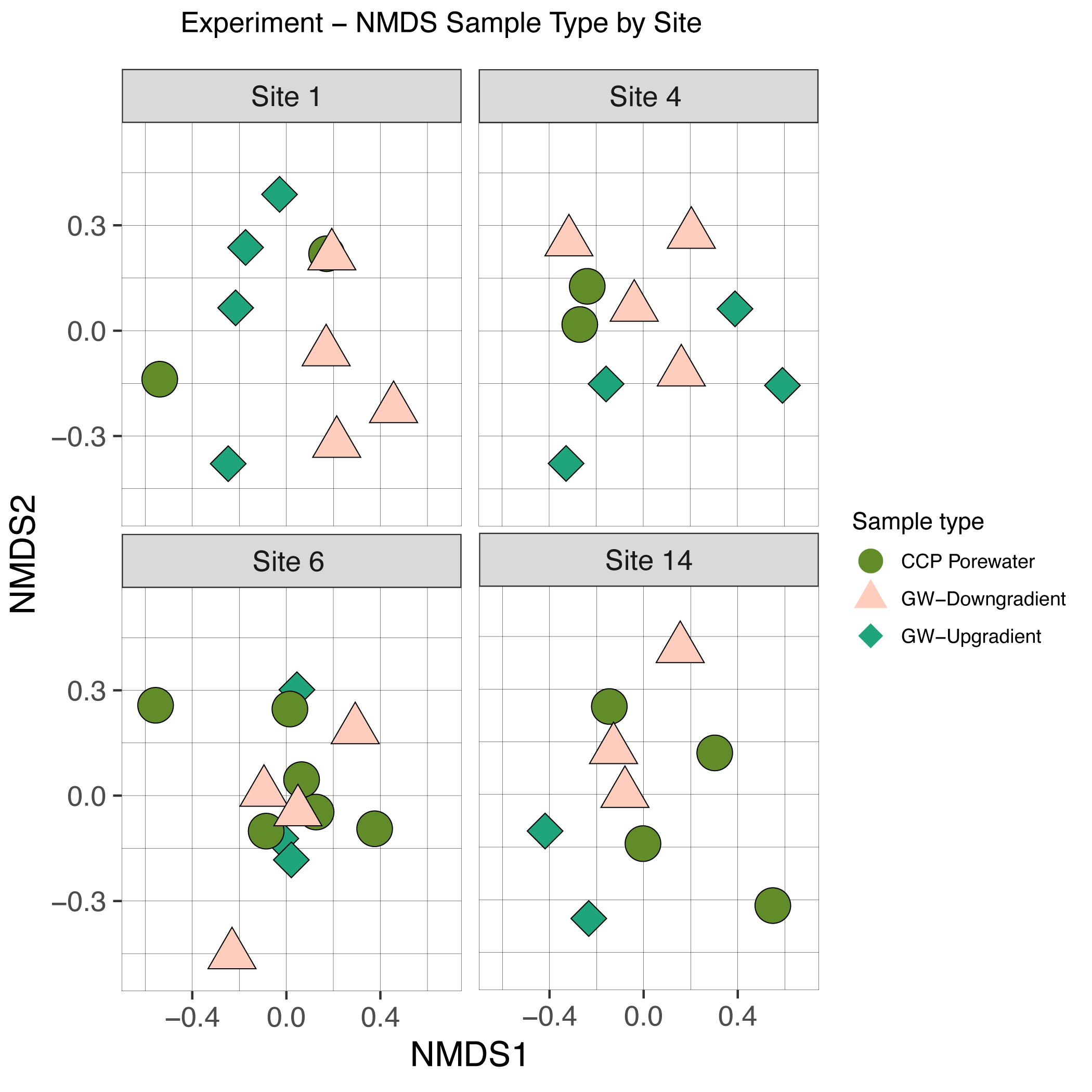

Supplement: SUPPLEMENTARY FIGURE 4 — Individual NMDS plots of ASV relative abundance for subset of sites that have enough samples for statistical analyses. Each NMDS represents a single site, and within the site, shapes and colors denote the sample types. Note that upgradient groundwater plots within downgradient groundwater and CCP porewater samples for sites 3586, 17481, and 22354; while it plots separately (to the left side) for site 46001. [file Image_4.TIF]

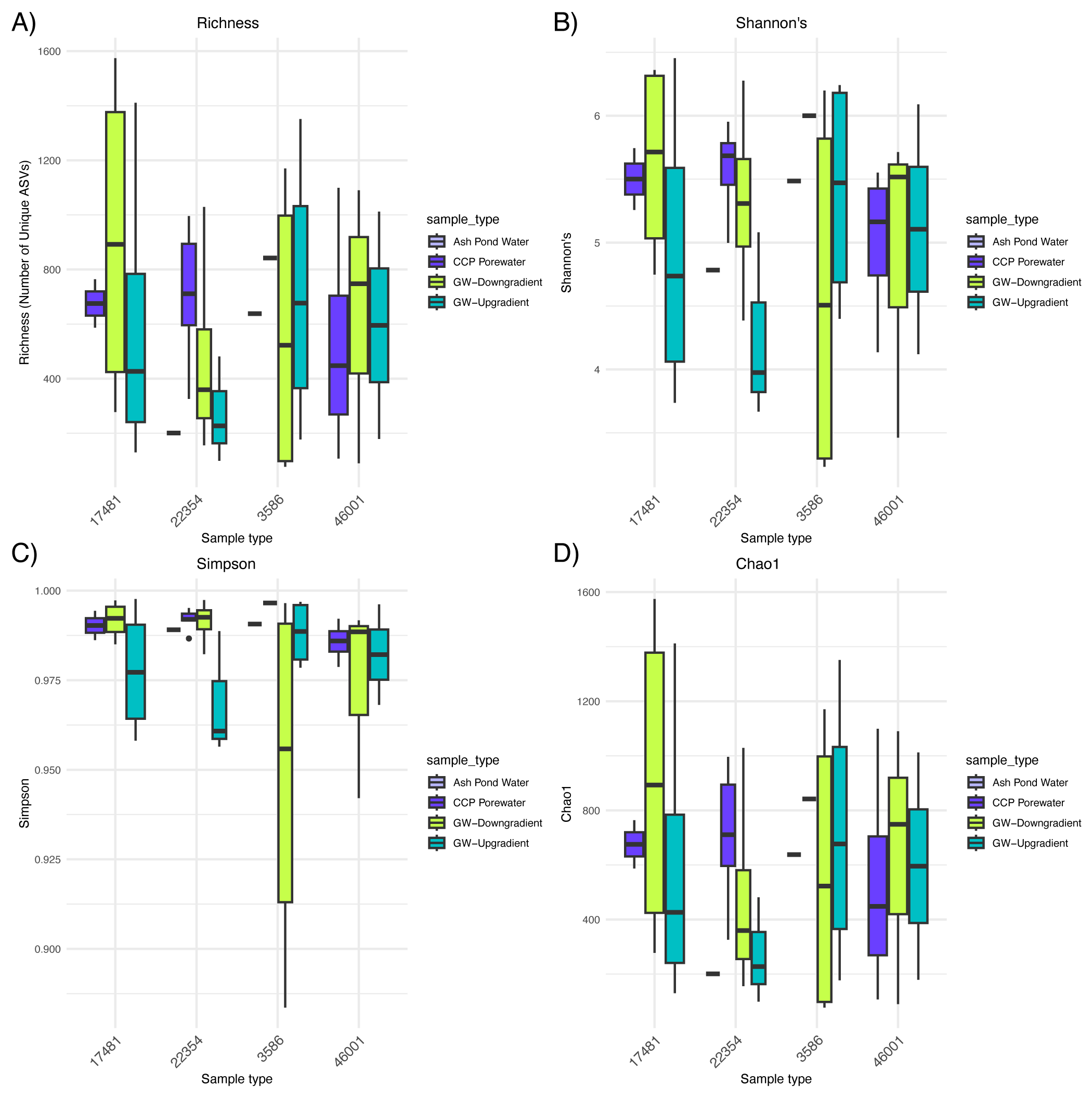

Supplement: SUPPLEMENTARY FIGURE 5 — ASV richness metrics across subset of sites that had >6 samples per sample type. Boxes denote the overall richness by either: (A) total unique counts of ASVs, (B) Shannon’s diversity, (C) Simpson’s diversity, (D) Chao diversity. [file Image_5.TIF]

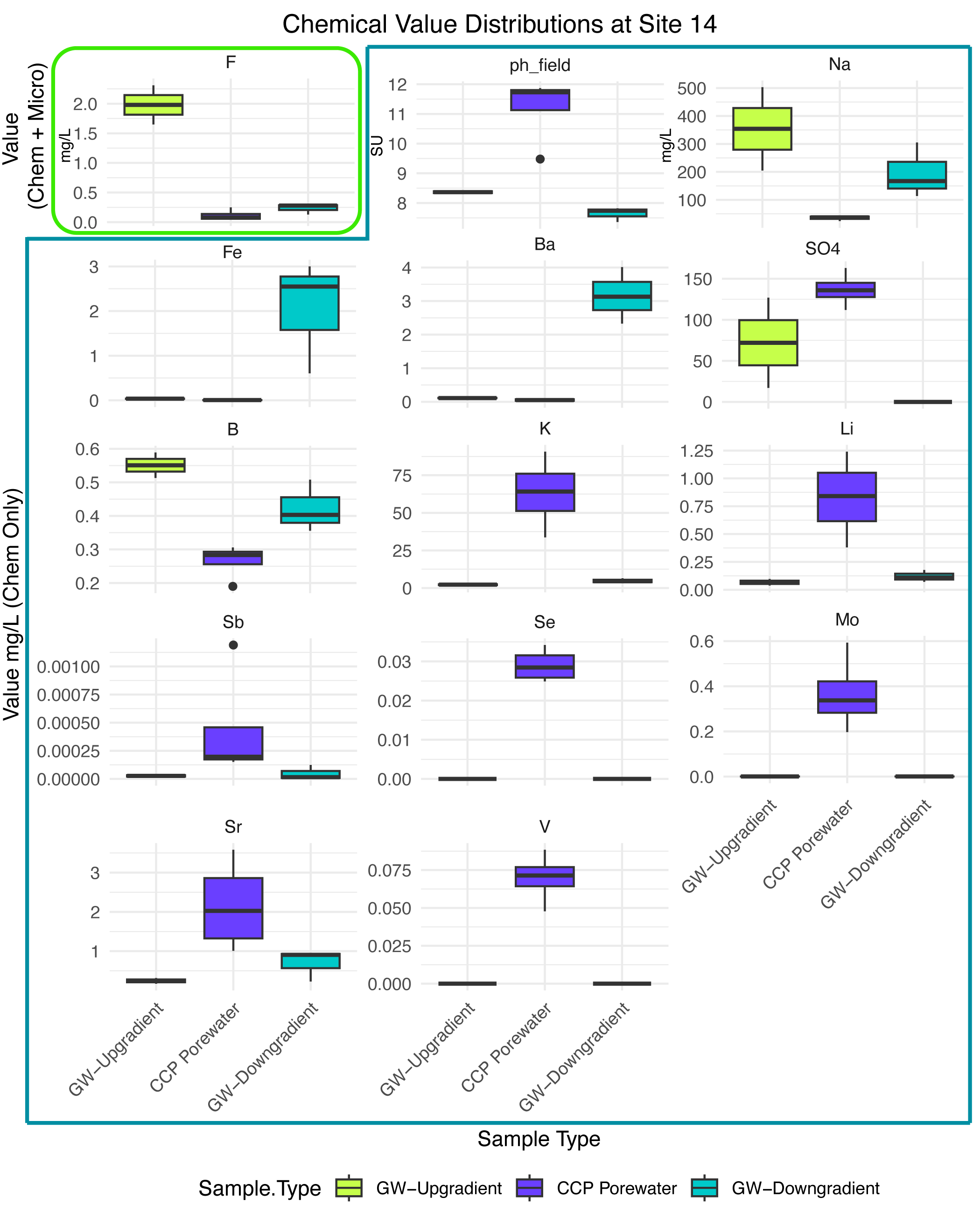

Supplement: SUPPLEMENTARY FIGURE 6 — Chemistry that was significantly different across sample types for Site 14. Box plots denote each significantly different chemical identified by a Kruskal-Wallis test at Site 14. Green box denotes chemistry that was significantly different by both microbial and chemical data. The blue box denotes chemistry that was identified as significant by only chemical data. [file Image_6.TIF]
